# ﻿Taxonomic review of the Oriental genus *Polyplocia* Lestage, 1921 (Ephemeroptera, Euthyplociidae), with two new records for Thailand

**DOI:** 10.3897/zookeys.1179.107312

**Published:** 2023-09-11

**Authors:** Sedtawut Kwanboon, Boonsatien Boonsoong, Chanaporn Suttinun

**Affiliations:** 1 Animal Systematics and Ecology Speciality Research Unit (ASESRU), Department of Zoology, Faculty of Science, Kasetsart University, Bangkok 10900, Thailand; 2 Biodiversity Center Kasetsart University (BDCKU), Bangkok 10900, Thailand; 3 Department of Veterinary Bioscience and Veterinary Public Health, Faculty of Veterinary Medicine Chiang Mai University, Chiang Mai 50100, Thailand; 4 Research Center for Veterinary Biosciences and Veterinary Public Health, Faculty of Veterinary Medicine Chiang Mai University, Chiang Mai 50100, Thailand

**Keywords:** Burrowing mayfly, Continental Asia, distribution, habitat, Insular Asia, taxonomy

## Abstract

Previously, the euthyplociid mayfly from Thailand was reported as *Polyplocia* spp. without designation to any nominal species. In this study, the genus *Polyplocia* Lestage, 1921 in Thailand was reviewed. Two species are recognized: *Polyplociaorientalis* Nguyen & Bae, 2003 and *P.nebulosa* Gonçalves & Peters, 2016. This is the first report of *P.nebulosa* in Continental Asia. Cytochrome c oxidase subunit I (COI) data and illustrations based on nymphal characters were used to confirm two different species in Thailand. Additional morphological characteristics of the nymph and eggs of the two species from Thailand are also shown in detail. The taxonomic status of *Polyplocia* in the Oriental region is reviewed. Keys to known nymphal and imaginal stages are provided and the distribution of *Polyplocia* in the Oriental region is discussed.

## ﻿Introduction

Lestage (1921) established the genus *Polyplocia*, and *Polyplociavitalisi* Lestage, 1921 from Tonkin, Vietnam, was considered the type species based on one male imago. Ulmer (1939) described two new species *P.campylociella* Ulmer, 1939 and *P.crassinervis* Ulmer, 1939 based on a northern Borneo (now Sabah, Malaysia) subimago. Demoulin (1952) revised the imago characters of *Polyplocia* as having a transverse pronotum, forewing with MA fork at same level as Rs fork, and at least one intercalary vein in the cubital field with its base connected to CuP, forceps with only one segment and three caudal filaments on the abdomen (Gonçalves and Peters 2016) and provided new figures of *P.crassinervis* and *P.vitalisi*. Demoulin (1953) later described the female imago, also from Sarawak, and synonymized the species as *P.campylociella* (= *P.crassinervis*) based on wing venation. He later classified a potential nymph of *Polyplocia* for the first time from West Borneo (now Indonesia) as *P. ?crassinervis*, despite the synonymy, and identified the nymphal characters: long tusks with truncated apex, antennae almost the same length as the tusks, foretibiae with short apical projection, foretarsi with apical projection, and gills I with two lamellae (Demoulin 1966). Thirty-seven years later, Nguyen and Bae (2003a) described a new species, *P.orientalis* Nguyen & Bae, 2003, based on nymphs from Dak Lak, Vietnam and stated that this nymph could be associated with *P.vitalisi*. Gonçalves and Peters (2016) discovered a new species, *P.nebulosa* Gonçalves & Peters, 2016, based on male and female imagos from Malaysia’s Sabah State, and provisional nymphs assigned to this species are also described. A description of the structure of the chorion was also provided and recommended as a useful tool for recognising species (Gonçalves and Peters 2016). Lastly, Zheng et al. (2023) reported the first nymph-imago association for the genus in *P.orientalis* from China and expanded the distribution of this species in the Oriental region. Molecular evidence based on cytochrome c oxidase subunit I (COI) revealed an intraspecific distance of 10% between the Vietnamese and Chinese specimens.

To date, the genus *Polyplocia* is composed of four valid species: *P.vitalisi* reported only in an adult stage, *P.campylociella* reported in adult stage with possible nymphal stage, *P.orientalis* reported in both stages and *P.nebulosa* reported in adult stage with a possible nymphal stage. The distribution of this genus encompasses the Oriental Realm (Lestage 1921; Ulmer 1939; Demoulin 1966; Nguyen and Bae 2003a; Gonçalves and Peters 2016; Zheng et al. 2023). *Polyplocia* spp. nymphs from Thailand were mentioned by Kluge (2004) and Gonçalves and Peters (2016). These nymphs may be related to *P.orientalis* or belong to another species, as they did not possess paired black anterolateral marks on abdominal sterna. Conversely, the genitalia of the male nymphs of *Polyplocia* spp. from Thailand also had T-shaped penes, as do the nymphs of *P.nebulosa* (Gonçalves and Peters 2016). In the present study, two species of the genus *Polyplocia* based on nymphs from Thailand are recorded using morphological and molecular approaches. Some additional descriptions on the nymphal and chorion structures are mentioned. Keys to known species in both stages are provided. The distribution of this genus in Thailand is studied and information on the biological aspects of *Polyplocia* is provided.

## ﻿Materials and methods

The specimens were collected by hand-picking from headwater streams in the northern and the western parts of Thailand. (Table 1, GPS map versatile navigator (Garmin eTrex 10)). The specimens were preserved in 100% ethanol for molecular and morphological studies. The mature nymphs were reared using earthenware pots connected to an oxygen pump until emergence of winged stages. Measurements (given in mm) and photographs were taken using a NIKON SMZ800 stereoscopic microscope. For scanning electron microscopy (SEM), eggs were dried in a critical point drier (CPD7501) and coated with gold (Sputter Coater SC7620). The SEM photographs were obtained with a FEI Quanta 450 SEM. Final plates were prepared with Adobe Photoshop CC 2022.

**Table 1.** GPS coordinates of locations of examined specimens.

Species	Locality	GPS coordinates
*Polyplociaorientalis*	Chiang Rai (CR)	19°26'53.7"N, 99°41'83.6"E
	Chiang Mai (CM)	19°19'31.1"N, 99°58'84.6"E
*Polyplocianebulosa*	Kanchanaburi (KN)	14°33'10.8"N, 98°33'94.3"E
	Phetchaburi (PE)	12°28'53.2"N, 99°15'23.0"E
*Polyplociacampylociella*	Sarawak, Malaysia	4°02'51.0"N, 114°50'11.9"E

Selected specimens were dissected for DNA extraction. Total DNA was extracted using a genomic DNA purification kit (NucleoSpin, Macherey-Nagel, Germany), following the manufacturer’s protocol. The COI amplification was performed using LCO1490 and HCO2198 (Folmer et al. 1994). The polymerase chain reaction (PCR) conditions and procedure were as described by Kwanboon et al. (2021). The PCR products were purified using a Gel and PCR Clean-up Kit (NucleoSpin, Macherey-Nagel, Germany) and were sequenced by ATGC Co., Ltd (Thailand). Other analysed *Polyplociaorientalis* sequences were obtained from GenBank (OP347109; OP962407). *Potamanthusformosus* Eaton, 1892, retrieved from GenBank (MZ453438), was used as an outgroup. The genetic distances between species were determined using Kimura-2-parameter distances (Kimura 1980), calculated with the MEGA11 program (Tamura et al. 2021). Sequence alignment and editing were performed using ClustalW in MEGA11. A phylogenetic tree was analysed by the maximum likelihood (ML) method and the most appropriate evolutionary model was calculate using the Find Best DNA/Protein Models (ML) option test provided with MEGA11. The Tamura-Nei 93 model and a proportional discrete Gamma distribution (TN93+G) was performed with MEGA11 using the likelihood-ratchet method with 1000 bootstrap replicates. The GenBank accession numbers are given in Table 2, nomenclature of gene sequences follows Chakrabarty et al. (2013). Nucleotide sequences obtained in this study have been deposited in GenBank. The distribution map was generated with the SimpleMappr software (https://simplemappr.net; Shorthouse 2010).

**Table 2.** Sequenced specimens of the genus *Polyplocia*.

Species	Locality	Code	GenBank #	GenSeq Nomenclature
*P.nebulosa*	Kanchanaburi, Thailand	PC02KN	OR366860	genseq-4 COI
	Phetchaburi, Thailand	PC01PE	OR366862	genseq-4 COI
	Kanchanaburi, Thailand	PC08PE	OR366863	genseq-4 COI
*P.orientalis*	Chiang Rai, Thailand	PC04CR	OR366857	genseq-4 COI
	Chiang Rai, Thailand	PC05CR	OR366858	genseq-4 COI
	Chiang Rai, Thailand	PC06CR	OR366861	genseq-4 COI
	Chiang Mai, Thailand	PC01CM	OR366859	genseq-4 COI
	Vietnam		OP347109	
	Yunnan, China		OP962407	
*P.campylociella*	Sarawak, Malaysia		OR366864	genseq-4 COI

The material was deposited in the collection of the Zoological Museum at Kasetsart University in Bangkok, Thailand (ZMKU) and the Veterinary Anatomy and Pathology Museum, Chiang Mai University, Thailand (VMCMU).

We followed all guidelines of the Animal Ethics Committee of Kasetsart University (approval no. ACKU61-SCI-029) for collecting the mayfly specimens.

## ﻿Results

### ﻿Taxonomic review and additional description of nymphal stage

**Figure 1.**
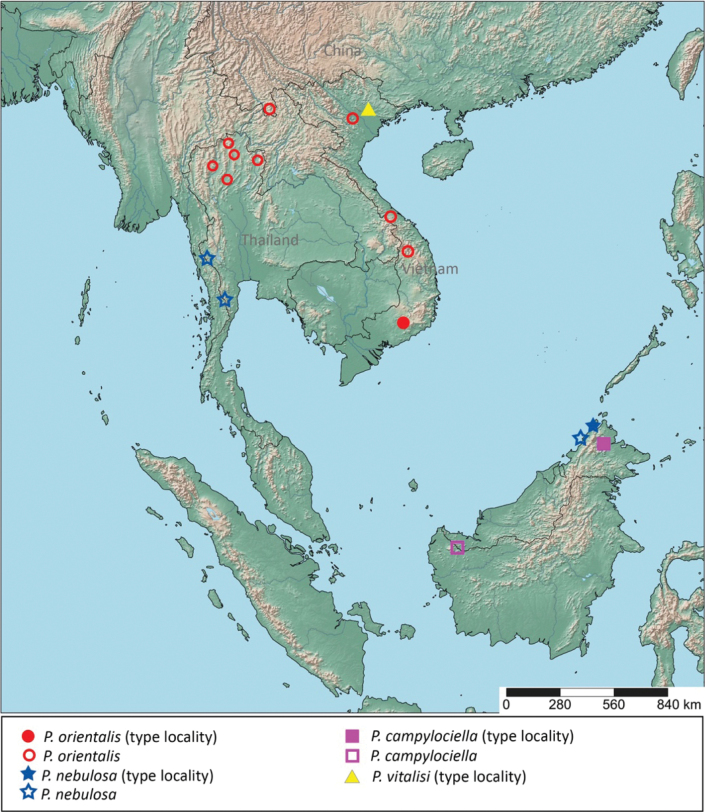
Distribution map of the genus *Polyplocia* in the Oriental region.

Nymphs of *Polyplocia* spp. from northern of Thailand of Gonçalves and Peters (2016) are assigned to *P.orientalis*. The additional description of *P.orientalis* is based on material from the northern of Thailand (Chiang Rai and Chiang Mai provinces) and variations regarding these populations are given. Five nymphs of *P.nebulosa* are recorded from western Thailand (Kanchanaburi and Phetchaburi provinces). A map of *Polyplocia* species distribution in Oriental region is given in Fig. 1.

### ﻿Order Ephemeroptera Hyatt & Arms, 1891

**Family Euthyplociidae Edmunds & Traver, 1954**

#### ﻿Genus Polyplocia

Taxon classificationAnimaliaEphemeropteraEuthyplociidae

Lestage, 1921

1C8BC9CC-0C0F-58BB-B888-F1A0E0F8ABAC

Polyplocia Lestage, 1921: 212 (Type: Polyplociavitalisi); Lestage 1924: 5; Ulmer 1932: 205; Ulmer 1939: 466; Demoulin 1952: 9; Demoulin 1966: 137; Nguyen and Bae 2003a: 280; Gonçalves and Peters 2016: 553; Zheng et al. 2023: 2.

##### Diagnosis.

**Imago**: i) transverse pronotum, ii) forewing with MA fork at same level as Rs fork, and at least one intercalary vein in the cubital field with its base connected to CuP, iii) forceps with only one segment, and iv) three caudal filaments on abdomen (Demoulin 1952; Gonçalves and Peters 2016). **Nymph**: i) mandibles very long, with apex obliquely truncated, ii) antennae almost the same length as the mandibles, iii) anterior angles of the pronotum protruding into points iv) foretibia terminated by an apophysis provided with an internal brush, v) fore tarsi with a distal unguiform extension, vi) gill I bilamellated, and vii) bare terminal filament (Demoulin 1966; Gonçalves and Peters 2016: 554).

#### ﻿ Polyplocia vitalisi

Taxon classificationAnimaliaEphemeropteraEuthyplociidae

Lestage, 1921

6782F29E-716A-5906-9213-328C86846081

Polyplocia vitalisi Lestage, 1921: 212, original description (male imago).
Polyplocia vitalisi (Ulmer 1939: 467, figs 7–9, male imago).
Polyplocia vitalisi (Demoulin 1952: 16, fig. 3, male imago).
Polyplocia vitalisi (Gonçalves and Peters 2016: 558, male imago).

##### Material examined.

None.

##### Diagnosis.

**Imago**: i) transparent wing, ii) membrane of forewing with purplish colour on C and Sc fields, iii) abdominal sterna without marking, and vi) penis lobe with a smooth apical border (Lestage 1921).

##### Description.

**Male imago.** See Lestage (1921).

**Female imago.** Unknown.

**Nymph.** Unknown.

##### Distribution.

Tonkin (Vietnam).

##### Remark.

This species was described based on one male imago from Tonkin, Vietnam.

#### ﻿ Polyplocia campylociella

Taxon classificationAnimaliaEphemeropteraEuthyplociidae

Ulmer, 1939

627CA3DE-50D0-57F8-9981-81530FE75950

= P.crassinervis Ulmer, 1939 (Demoulin 1953).
Polyplocia campylociella Ulmer, 1939: 468, figs 10–11, original description (female subimago).
Polyplocia crassinervis Ulmer, 1939: 470, figs 12–15, male subimago).
Polyplocia crassinervis (Demoulin 1952: 18, fig. 4, male imago).
Polyplocia campylociella (Demoulin 1953: 1, fig. 1, female imago = P.crassinervis).
Polyplocia campylociella (Demoulin 1966: 137, fig. 1, immature nymph).
Polyplocia campylociella (Gonçalves and Peters 2016: 558, male and female imago).

##### Material examined.

**Malaysia**: One immature nymph in alcohol, deposited in ZMKU, Sarawak, Marudi district, Miri division, Gunung Mulu, Sungai Paku, 4°02'51.0"N, 114°50'11.9"E, ~240 m, 13.VI.2023, B. Boonsoong leg.

##### Diagnosis.

The imago of *P.campylociella* can be distinguished from those of other *Polyplocia* species based on the following characteristics: i) wings with dark clouds around cross veins and margins, ii) membrane of forewing with little dark colour on C and Sc fields, iii) styliger plate rounded, and iv) penis V-shaped without dorsal spine (Demoulin 1952).

##### Description.

**Male imago.** See Demoulin (1952).

**Female imago.** See Demoulin (1953).

**Nymph.** See Demoulin (1966). In this study, only one immature nymph was collected. The specimen possessed a pair of anterolateral black marks on abdominal sterna. COI sequences were analysed from the sample.

##### Distribution.

Malaysia (Sarawak), Indonesia (Sambas).

##### Remarks.

The possible nymph of *P.campylociella* was described based on one immature nymph by Demoulin (1966) from Sambas (Indonesia). Additional illustrations of *P.campylociella* are provided in Miller et al. (2018) and Jacobus et al. (2019). The distribution of *P.campylociella* is limited to Insular Asia.

#### ﻿ Polyplocia nebulosa

Taxon classificationAnimaliaEphemeropteraEuthyplociidae

Gonçalves & Peters, 2016

09A7B00C-3978-5511-B894-15EE9C5E3465

Figs 2 , 3

Polyplocia nebulosa Gonçalves & Peters, 2016: 554, figs 1–21, original description (male and female imago, female subimago, egg, possible nymph).

##### Material examined.

**Thailand**: Two nymph in alcohol, deposited in ZMKU, Kanchanaburi province, Thong Pha Phum district, Pat Sadu Klang, 14°33'10.8"N, 98°33'94.3"E, 349 m, 20.II.2016, B. Boonsoong leg. Three nymphs in alcohol, deposited in ZMKU, Phetchaburi province, Kaeng Krachan district, Ban Krang river, 12°28'53.2"N, 99°15'23.0"E, 386 m, 11.II.2023, A. Vitheepradit leg.

##### Diagnosis.

**Imago**: i) wings with longitudinal veins light brown and cross veins brown, cross veins with narrow dark brown clouds and margins tinged with brown, ii) sterna II–IX with a pair of blackish brown anterolateral marks, iii) penes broad, T-shaped, fused, with medial groove extending from apex to half-length of penes; large laterally projecting lobes apically rounded with a small dorsolateral spine; basal outer margin of each lobe sclerotized, iv) styliger plate short and straight, not projected posteriorly, and v) eggs 265–267 μm in length and 170–186 μm in width, barrel shaped, without polar caps or other attachment structures, with one visible micropyle and chorion forming an irregular mesh with raised ridges, mesh size from 3.8–8.0 μm (Gonçalves and Peters 2016). **Nymph**: i) larger spines on distal ½ of tusk, and ii) sterna with a pair of anterolateral black marks (Gonçalves and Peters 2016).

##### Description.

**Male imago.** See Gonçalves and Peters (2016).

**Female imago.** See Gonçalves and Peters (2016).

**Nymph.** See Gonçalves and Peters (2016).

##### Additional description.

Mandibular tusks (Fig. 3E) strongly arched inward (20.7° in curvature, for angle measurement see Kwanboon et al. (2021)). Labrum (Fig. 3A): median concave with shallow emargination, with long and short setae on sub-basal, subapical and anterior margins. Hypopharynx (Fig. 3B): lingua cordiform, superlingua slightly extended laterally with dense setae on distal margin. Labium (Fig. 3C): paraglossae with dense setae on ventral margin, meet above glossae; glossae drop-shaped; palpi with long setae on outer margin; 3^rd^ segment much broader, acutely rounded at the apex. Maxilla as in Fig. 3D: 1^st^ segment with a few fine setae on outer margin; 2^nd^ segment with long hair-like setae in both inner and outer margin; 3^rd^ segment long, at least 2× longer than 2^nd^ segment, apically pointed with numerous long, hair-like setae.

**Figure 2.**
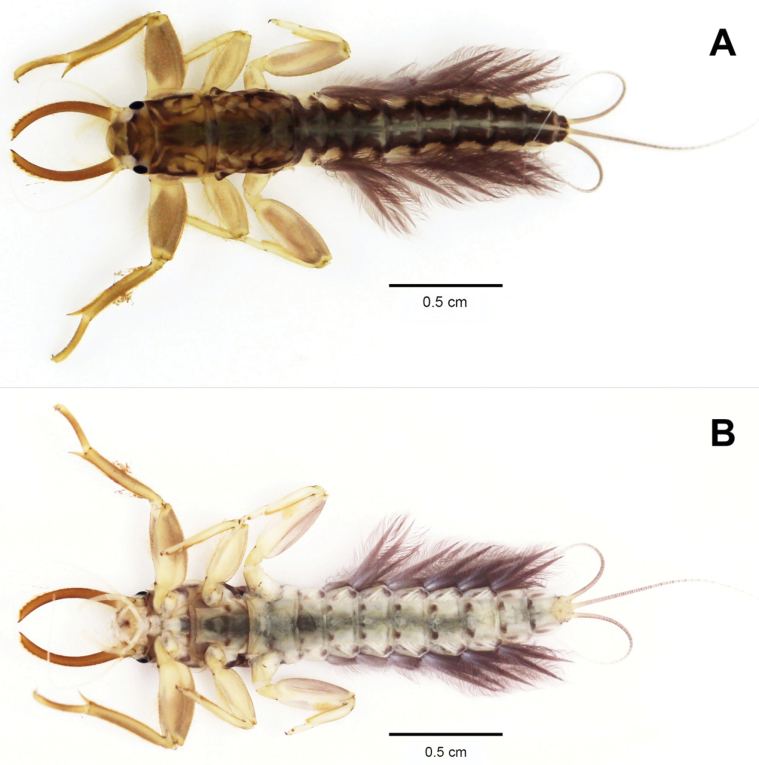
Habitus of nymph of *Polyplocianebulosa*: **A** dorsal view **B** ventral view.

**Figure 3.**
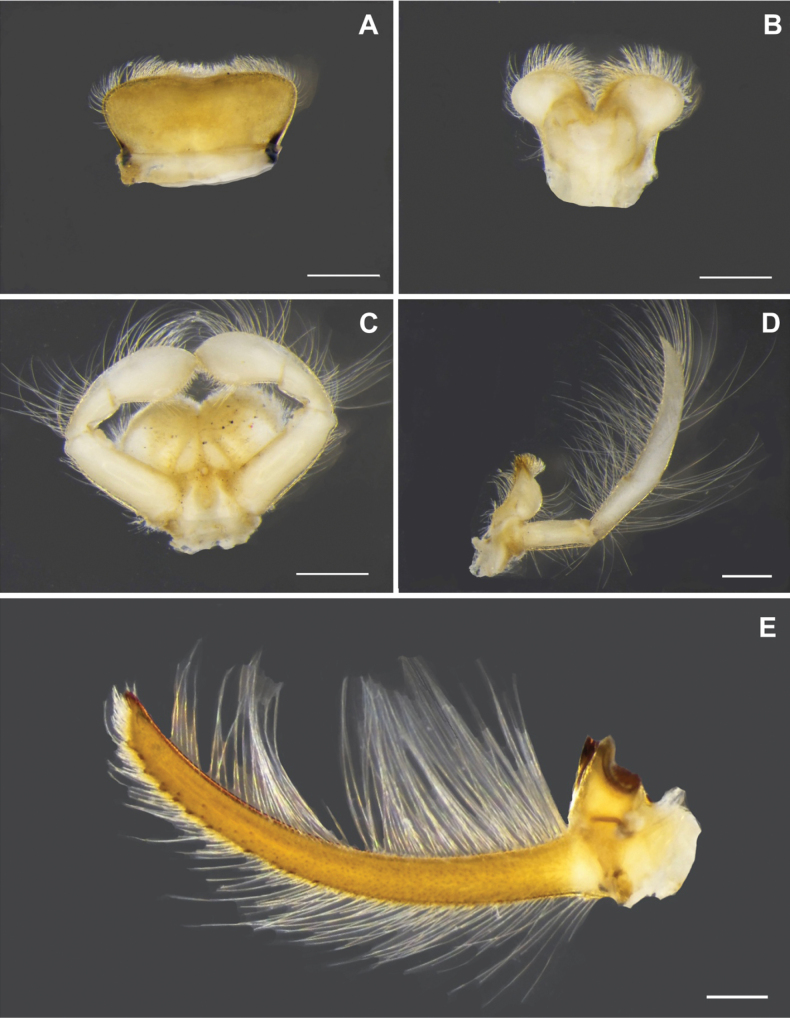
*Polyplocianebulosa*, nymphal morphology: **A** labrum (dorsal view) **B** hypopharynx (ventral view) **C** labium (ventral view) **D** left maxilla (ventral view) **E** mandibular tusk. Scale bar: 0.5 mm.

##### Distribution.

Malaysia (Sabah), Thailand (Kanchanaburi, Phetchaburi).

#### ﻿ Polyplocia orientalis

Taxon classificationAnimaliaEphemeropteraEuthyplociidae

Nguyen & Bae, 2003

29CF4B22-8A17-52A6-A20A-2A2817A60108

Figs 4 , 5 , 6

Polyplocia orientalis Nguyen & Bae, 2003a: 280, figs 1–2, original description (nymph).
Polyplocia orientalis (Gonçalves and Peters 2016: 558, nymph).
Polyplocia spp. (Gonçalves and Peters 2016: 558, possible nymph (Thailand)).
Polyplocia orientalis (Zheng et al. 2023: 2, figs 1–9, male and female imago, egg, nymph).

##### Material examined.

**Thailand**: Eleven nymphs in alcohol, deposited in ZMKU, Chiang Rai province, Phan district, Pu Kaeng waterfall, 19°26'53.7"N, 99°41'83.6"E, 540 m, 5.III.2021, S. Kwanboon leg. Three nymphs in alcohol, deposited in VMCMU, Chiang Rai province, Phan district, Pu Kaeng waterfall, 19°26'53.7"N, 99°41'83.6"E, 540 m, 29.I.2023, S. Chanaporn leg. Two nymphs in alcohol, deposited in ZMKU, Thailand, Chiang Mai province, Chiang Dao district, Huay Mae Mae, 19°19'31.1"N, 99°58'84.6"E, 809 m, 20.XI.2018, C. Damrong leg.

##### Diagnosis.

**Imago**: i) membrane of wings transparent and colourless, and ii) T-shaped male penis with apical depression on both lobes (Zheng et al. 2023). **Nymph**: i) large body size (25.0–46.4 mm), ii) yellowish abdominal sterna without anterolateral black mark, iii) spines on 1/3 of tusk length distally, and iv) apically expanded dorsal lobe of gill I (Nguyen and Bae 2003a; Zheng et al. 2023).

##### Description.

**Male imago.** See Zheng et al. (2023).

**Female imago.** See Zheng et al. (2023).

**Nymph.** See also Nguyen and Bae (2003a) and Zheng et al. (2023).

##### Variability and additional description

**(Thai specimens). Nymph.** Male body length 17.22 mm; cerci length 11.78 mm; median filament length 10.5 mm. Female body length 27.9 mm; cerci length 18.5 mm; median filament length 16.2 mm.

***Head*.** Length 2× of maximum width; narrower than pronotum. Compound eye black on dorsolateral margin. Antenna 8.8 mm in length; scape with at least three short setae.

**Figure 4.**
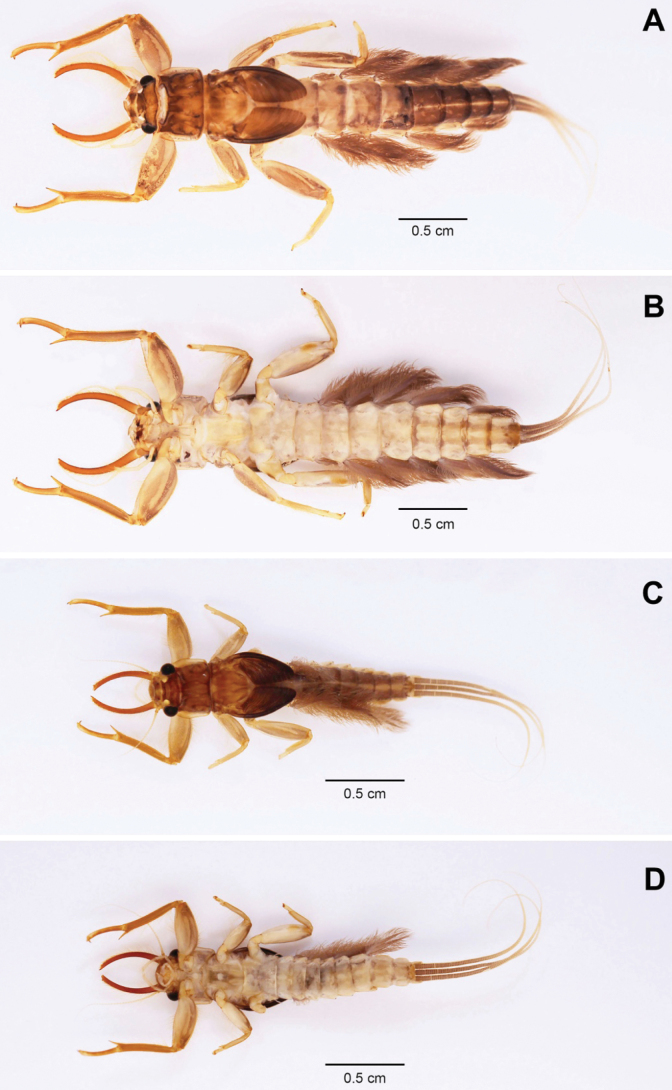
Habitus of nymph of *Polyplociaorientalis*: **A** female mature nymph (dorsal view) **B** female mature nymph (ventral view) **C** male mature nymph (dorsal view) **D** male mature nymph (ventral view).

**Figure 5.**
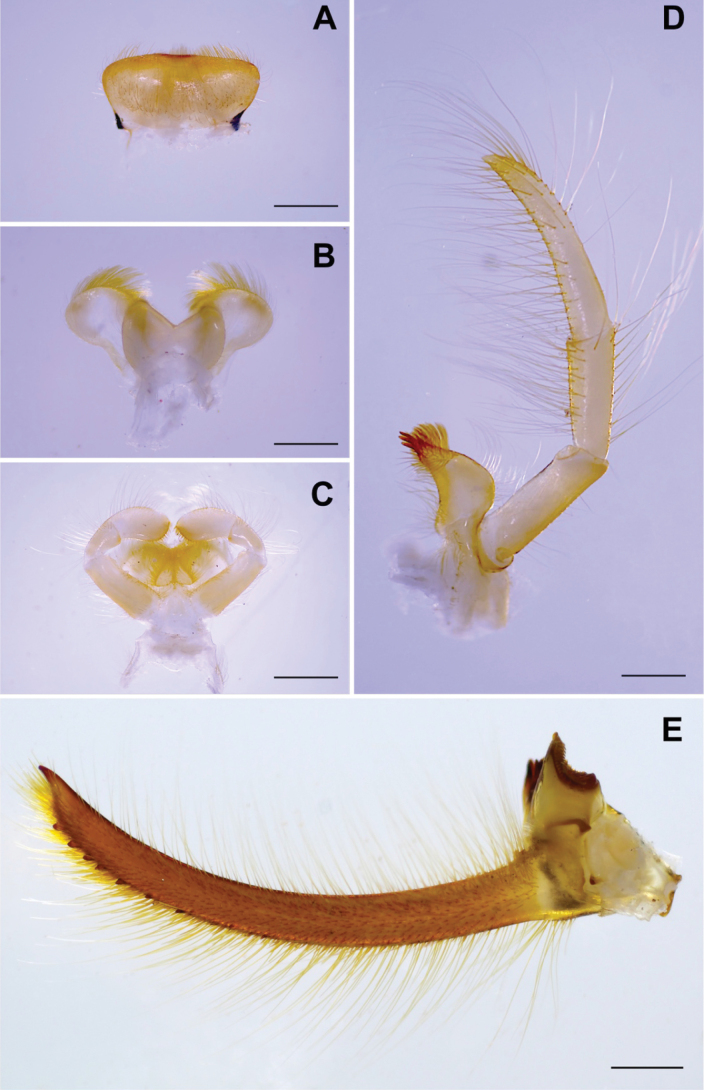
*Polyplociaorientalis*, nymphal morphology: **A** labrum (dorsal view) **B** hypopharynx (ventral view) **C** labium (ventral view) **D** left maxilla (ventral view) **E** mandibular tusk. Scale bar: 0.5 mm.

***Mouthpart*.** Labrum (Fig. 5A) shallow, concave at anteromedian margin, with a tuft of dense long, simple setae; anterior margin with a row of 14–16 long, simple setae; dorsal surface with long, fine setae and short, simple setae scattered over area. Mandibular tusks (Fig. 5E) strongly arched inward (18.6° in curvature); a row of simple setae on base of tusks; ventrally almost bare. Right mandible without prostheca. Left mandible with prostheca as long as incisors, truncate, broader apically. Maxillary palp (Fig. 5D) 1^st^ segment with a few of long, fine setae on outer margin; 2^nd^ segment with lateral long, hair-like setae in both inner and outer margin; 3^rd^ segment long, at least 6 × longer than wide, apically pointed, with numerous of long, hair-like setae on lateral inner margin and scattered over half of segment apically. Hypopharynx (Fig. 5B) lingua cordiform; superlingua extended laterally. Labium (Fig. 5C) paraglossae articulate above glossae; labial palpi 1^st^ segment with long, hair-like setae on outer margin and fine, simple setae on inner margin; 2^nd^ segment with long, hair-like setae on outer margin; 3^rd^ segment much broader than 2^nd^ segment, apically truncated almost straight with a tuft of stout, simple setae, with long, hair-like setae on outer margin and fine, simple setae on inner margin.

***Legs*.** Ratio of forelegs: midlegs: hindlegs 1:0.7:0.7; ratio of foreleg segments 1:1.2:0.7:0.4; ratio of midlegs segments 1:1:0.4:0.2 with moderately developed setae; ratio of hindleg segments 1:0.6:0.2:0.1, lack setae on femora.

**Figure 6.**
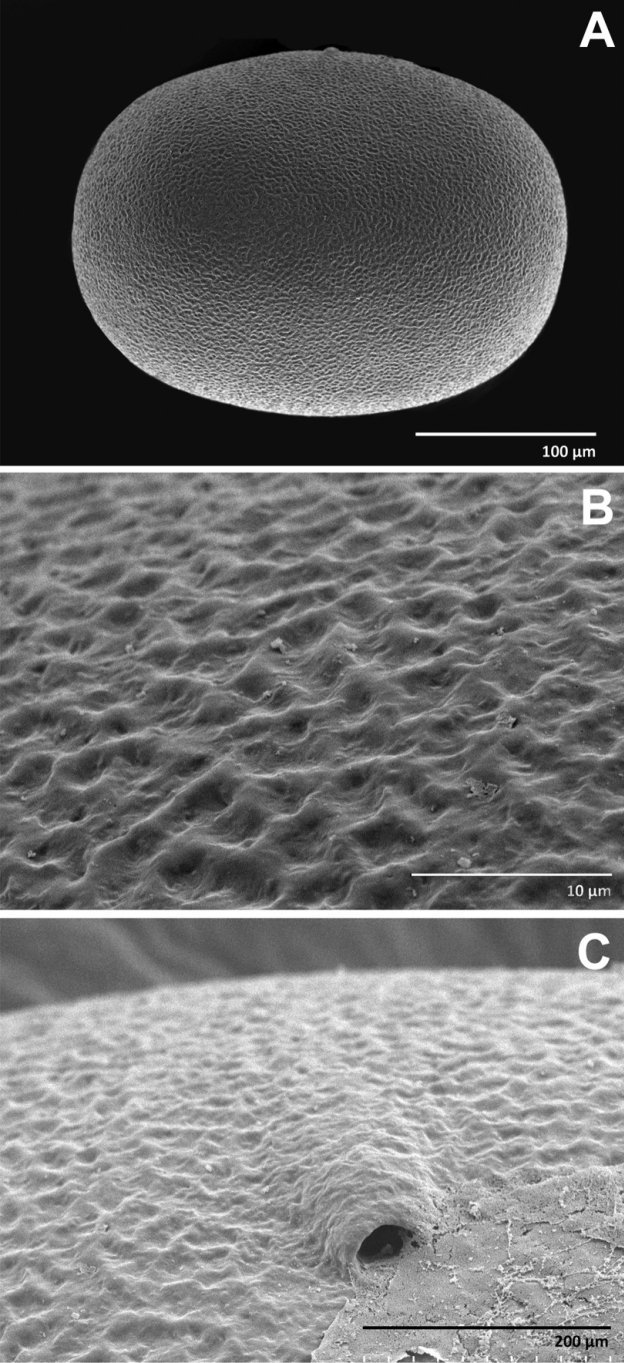
*Polyplociaorientalis*, SEMs of egg structures: **A** general outline of egg **B** chorion surface **C** micropyle.

**Female subimago. *Egg.*** (Fig. 6) Dissected from female subimago; length 272 μm, width 214 μm; oval-shaped; no polar caps or other attachment structures; rough chorionic surface, mesh-like with irregular raised ridges (Fig. 6B); two visible linear micropyles formed with micropyle canal on the surface (Fig. 6C).

In this study, Thai *Polyplociaorientalis* showed some variation in these characters combined: i) Labrum slightly concave on anterior margin, nearly straight, ii) tusks cylindrical pointed apically, strongly arched inward (18.6° curvature) and outer margin with 7–9 larger spines on 1/3 of tusk length distally, and iii) eggs oval-shaped with two visible micropores.

##### Distribution.

China (Yunnan), Vietnam (Dak Lak, Lam Dong, Thua Thien Hue), Thailand (Chiang Rai, Chiang Mai, Phrae, Phayao, Nan) (Nguyen and Bae 2003a, Gonçalves and Peters 2016, Zheng et al. 2023, this study).

### ﻿Key to mature nymphs of *Polyplocia*

**Table:** 

1	Abdominal sterna without a pair of anterolateral black marks (Fig. 4B, D)	***P.orientalis* Nguyeun & Bae, 2003**
–	Abdominal sterna with a pair of anterolateral black marks (Fig. 2B)	*P.nebulosa* Gonçalves & Peters, 2016 / *P.campylociella* Ulmer, 1939*

*The details of nymph of *P.campylociella* are insufficient because of the limited description by Demoulin (1966) and immaturity of the specimen in this study. However, ratio of arrangement of spines on mandibular tusk vs. tusk length of mature nymph (Jacobus et al. 2019) seems different from *P.nebulosa*.

### ﻿Key to imagos of *Polyplocia* (modified from Gonçalves and Peters 2016 and Zheng et al. 2023)

**Table:** 

1	Veins with dark brown clouds around cross veins and margins of wings with brownish tinge; membrane of forewing without purplish color on C and Sc fields (Sc field may be a little darker); abdominal sterna with a pair of blackish marks on anterolateral margins	**2**
–	Veins without dark clouds around cross veins and wing margins translucent; membrane of forewing colored only on C and Sc fields, purplish; abdominal sterna without markings	**3**
2	Styliger plate rounded and projected, penis V-shaped, penes apparently without dorsal spine	***P.campylociella* Ulmer, 1939**
–	Styliger plate not projected, short and straight, penis T-shaped, each lobe of penes with small dorsal spine laterally directed	***P.nebulosa* Gonçalves & Peters, 2016**
3	Both penis lobes have an apical depression	***P.orientalis* Nguyen & Bae, 2003**
–	Penis lobes with smooth apical margin	***P.vitalisi* Lestage, 1921**

### ﻿Biological aspects

**Figure 7.**
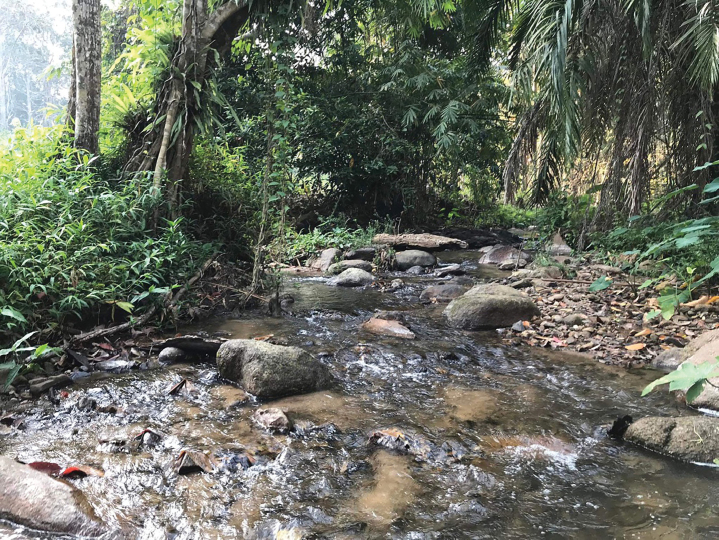
Habitat of *Polyplocianebulosa*.

**Figure 8.**
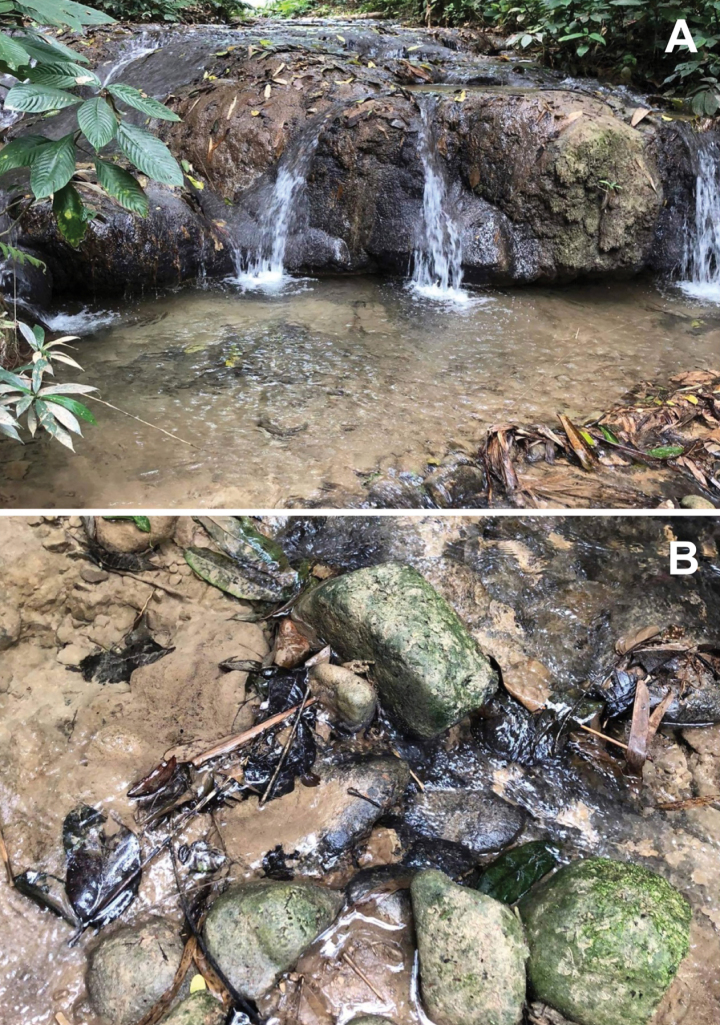
Habitat of *Polyplociaorientalis*: **A** Pu Kaeng waterfall (limestone waterfall) **B** microhabitat.

The genus *Polyplocia* was collected at an altitude of 300–800 metres above sea level; *Polyplocianebulosa* was found in a headwater stream in the forest, while *P.orientalis* was found in a headwater stream and a limestone waterfall quite disturbed by tourist activities. The nymphs were found on the underside of cobbles in slow-flowing waters at the margins of the stream (Figs 7, 8). The riverbed was covered in cobble, gravel and sand. The nymphs were frequently collected alongside the potamanthiid mayfly *Rhoenanthusmagnificus*.

### ﻿Molecular analysis

The partial sequences of the mitochondrial COI gene (658 bp) of the two species found in Thailand and *P.campylociella* from Malaysia were obtained from specimens of each locality (Table 2) to investigate the species delineation. The K2P analysis revealed that the intraspecific genetic distances of *P.orientalis* from Thailand vary between 0 and 10.7% and the intraspecific genetic distance of *P.nebulosa* from Thailand is 0.15–2.02%. The interspecific distances between Thai *P.orientalis* and *P.nebulosa* are high, ranging from 17.7–23.6% (Table 3). The sequences of four specimens of *P.orientalis* from Thailand and two sequences of *P.orientalis* from Vietnam and China present a K2P intraspecific distance ranging between 6.25% and 10.6%, respectively. The intraspecific distances of *P.orientalis* (Thailand) and *P.orientalis* (Vietnam) range between 9.51% and 10.6%, while *P.orientalis* (Thailand) and *P.orientalis* (China) range between 6.23% and 7.41%. The interspecific distances of all *P.orientalis* and *P.nebulosa* are 17.7%–23.6% and the interspecific distances of all *P.orientalis* and *P.campylociella* are 19.1%–21.7%, while the interspecific distances of *P.nebulosa* and *P.campylociella* are very high (20.7%–23.1%) (Table 3).

**Table 3.** Genetic distances (COI) between sequenced specimens using the Kimura 2-parameter.

Species		1	2	3	4	5
**1**	*P.orientalis* (Thailand)	0.00–10.7				
**2**	*P.orientalis* (China)	6.23–7.41	–			
**3**	*P.orientalis* (Vietnam)	9.51–10.6	10.0	–		
**4**	*P.nebulosa* (Thailand)	17.7–23.6	20.7–21.9	16.7–20.1	0.15–2.02	
**5**	*P.campylociella* (Malaysia)	19.1–20.6	19.8	21.7	20.7–23.1	–
**6**	*Potamanthusformosus*	22.8–24.1	23.8	23.0	23.0–24.3	25.2

**Figure 9.**
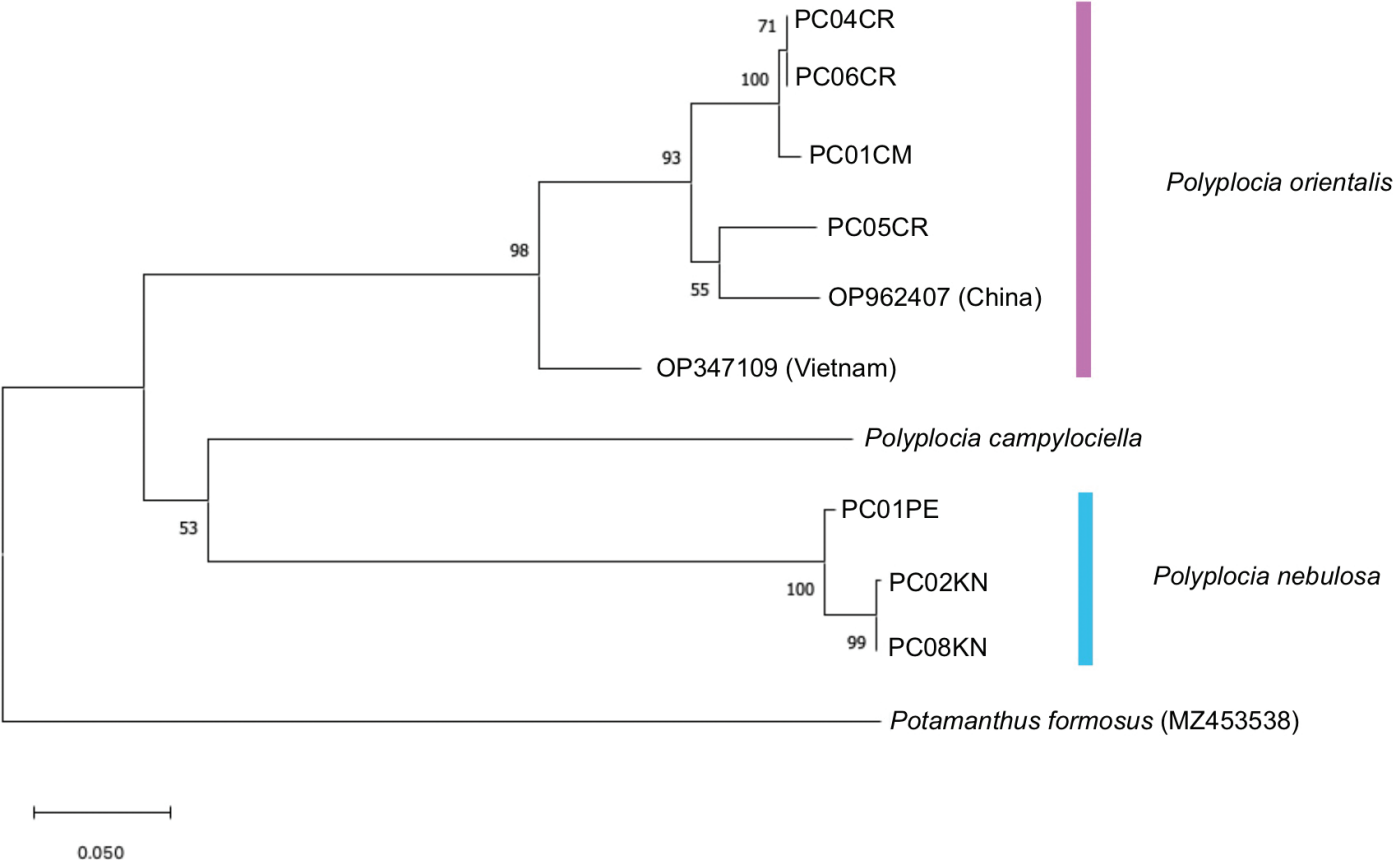
The COI phylogenetic construction based on the maximum likelihood (ML) analysis of three *Polyplocia* species, *Potamanthusformosus* was used as the outgroup. Scale bars refer to average of base substitution per site.

COI sequences analysis was built by maximum likelihood (ML) using MEGA11 (Fig. 9). Ten sequences of *Polyplocia* were separated into two main distinct clades: the first is the *P.nebulosa* and *P.campylociella* clade which is further separated into two clades of *P.nebulosa* (Thailand) and *P.campylociella* (Malaysia), while the second is a *P.orientalis* clade which is further separated into two clades of *P.orientalis* (Vietnam) and *P.orientalis* (Thailand, China).

## ﻿Discussion

Specimens of the *Polyplocia* from Thailand were studied based on the following nymphal characters defined by Demoulin (1966). Of the nominal species that are newly reported to the country, *P.orientalis* is distributed in northern Thailand (Chiang Rai and Chiang Mai provinces). Some variations in the morphological characters of the Thai *P.orientalis* were observed in this study (Table 4): Labrum slightly concave on anterior margin, nearly straight; the mandibular tusks are cylindrical, pointed apically and strongly arched inward (18.6° in curvature), with 7–9 spines on 1/3 of the tusk length distally. The second species, *P.nebulosa*, is distributed in western Thailand (Kanchanaburi and Phetchaburi provinces). Nymphs of these two species from Thailand can be easily separated by the presence of anterolateral black marks ventrally on *P.nebulosa* abdomen and their absence in *P.orientalis*. As we have only a very limited description of *P.campylociella* nymphs, their differences are not discussed in this study. The combinations of characters used to differentiate nymphs of known species are listed in Table 4.

**Table 4.** Selected nymphal characters of known species of the genus *Polyplocia*.

Characters		*P.orientalis* Nguyen & Bae, 2003	*P.nebulosa* Gonçalves & Peters, 2016	*P.campylociella* Ulmer, 1939
Head	antenna length vs. tusk length	longer	longer	slightly longer
Labrum	anterior margin	medially slightly concave with shallow emargination	medially concave with shallow emargination	medially concave (from fig. 1b)
Mandibular tusks	length of tusks vs. length of head	2×–3×	2×	?
	inward curvature	18.6°–21.3°	20.7° (in this study)	?
	number of spines	3–12	8–9 (fig. 18 Gonçalves and Peters 2016; this study)	?
	presence of large spines	Apical 1/3	Apical 1/2	Apical 1/3 (Jacobus et al. 2019)
Legs	length of apical spine of the foretibiae vs. length of the foretarsi	1/3–1/4	less than 1/2, 1/3 (in this study)	?
Abdomen	sterna	whitish	a pair of anterolateral black marks	a pair of anterolateral black marks
Winged stage	associated	yes	possible nymph	possible nymph
	eggs shape (subimago)	oval; barrel	barrel	?
	eggs length vs. width	1.3×	1.6×	?
	number of micropores	2	1	?
Distribution		Vietnam, China, Northern Thailand	Borneo, Malaysia, Western Thailand	Borneo, Malaysia
References		Nguyen and Bae (2003a); Zheng et al. (2023); this study	Gonçalves and Peters (2016); this study	Demoulin (1966)

The egg structure of all *Polyplocia* species has a similar chorionic surface pattern that forms an irregular mesh with raised ridges; however, we found small differences between the eggs of Thai and Chinese specimens of *P.orientalis*. The specimen from Thailand is oval-shaped, with two visible micropores, while *P.orientalis* from China and Vietnam has a barrel-shaped egg with no mention of a micropore (Nguyen and Bae 2003a; Zheng et al. 2023). The egg of the Thai *P.orientalis* shows different characters than *P.nebulosa* in terms of shape; the length vs width ratio, and the number of visible micropores. Gonçalves and Peters (2016) mention that the structure of the chorion should be considered as a foundation for future study of the genus. The present study confirmed that the structure of the chorion is useful for recognising the species.

The first molecular study of the genus *Polyplocia* was conducted by Zheng et al. (2023). The intraspecific distance of *P.orientalis* from Vietnam and China was mentioned to be as high as 10%. In our study, the maximum intraspecific distance between the Thai *P.orientalis* was relatively high, at 11%. When we compared the intraspecific distance of specimens from Thailand and Vietnam (10–11%) and Thailand and China (6–7%), the result from the genetic point of view also supported assigning the *Polyplocia* from northern Thailand to *P.orientalis*, as the maximum intraspecific distance of all specimens of *P.orientalis* is 11%. The genetic distances between the *P.orientalis* from northern Thailand and the sequence of *P.nebulosa* from western Thailand support the separation of this genus in Thailand into two species due to the high interspecific distance that ranges between 18% and 24%. The genetic distances of all known sequences of *P.orientalis* (sequences from Vietnam and China were added) and *P.nebulosa* from western Thailand also confirmed the separation into two species, with an interspecific distance of 18–24%. The result of interspecific distances between *P.campylociella* from Malaysia and two species from the Continental Asia are relatively high at the maximum 22% for *P.orientalis* and 23% for *P.nebulosa*. Kwanboon et al. (2021) also reported a variation in the interspecific distance as high as 14–20% for congeneric burrowing mayflies in Southeast Asia. The ML tree consists of two main clades; the *P.nebulosa* and *P.campylociella* clades and a *P.orientalis* clade (Fig. 9). The *P.nebulosa* and *P.campylociella* clades could represent two closely related species since they share some morphological characteristics, such as an abdominal sternum with two blackish marks on the anterolateral edges and wings with dark clouds around the cross veins and margins (Table 4). We recommend that molecular studies (i.e., the COI gene) be included as a basis for future studies of all burrowing mayflies, based on our findings and the studies of other burrowing mayflies.

*Polyplocia* nymphs from Thailand were mentioned by Kluge (2004) as *Polyplocia* sp. (locality and character are unknown) and by Gonçalves and Peters (2016) as *Polyplocia* spp. from many localities in northern Thailand. In this study, we collected specimens from one of the localities in which *Polyplocia* spp. were found by Gonçalves and Peters (2016) (Chiang Rai Province, Doi Luang National Park, Namtok Pu Keang). Therefore, the *Polyplocia* spp. of Gonçalves and Peters (2016) are proposed to be *P.orientalis* like the northern Thailand *Polyplocia* from this study. Although, the *Polyplocia* sp. of Kluge (2004) is not assigned to either *P.nebulosa* or *P.orientalis*, as the data are still lacking.

The distribution of the genus *Polyplocia* Lestage, 1921 in the Oriental region is shown in Fig. 1. *Polyplociaorientalis* was reported in Vietnam (Dak Lak, Lam Dong and Thua Thien Hue) and China (Yunnan) and, in this study, in the northern part of Thailand (Chiang Rai, Chiang Mai, Phrae, Phayao and Nan). Therefore, this species has the potential to be assigned as the dominent species of *Polyplocia* in mainland Asia in the Oriental region. Surprisingly, the discovery of *P.nebulosa* in Thailand is the first report of this species from Continental Asia, as all other specimens were reported from East Malaysia (Sabah) or Insular Asia. This wide range of distribution of mayflies between Continental and Insular Asia has been reported in many families. For example, *Potamanthelluscaenoides* Ulmer, 1939 (Neoephemeridae) is known as a widespread mayfly in the Oriental region. This species has also been recorded from the Insular Asia, in Indonesia (Sumatra, Java, Bali, Lombok and Flores), Malaysia (Sabah and Sarawak) and the Philippines (Mindanao), and in Continental Asia, in Malaysia (the Malay peninsula), Thailand (Chiang Mai), India (western Ghat) and Vietnam (Dak Lak) (Bae and McCafferty 1998; Nguyen and Bae 2003b; Sartori et al. 2003; Selvakumar et al. 2015; Banazair and Christopher 2019). Such wide distribution is also observed for the burrowing mayfly Rhoenanthus (Rhoenanthus) speciosus Eaton, 1881 of the family Potamanthidae. This species was reported from the Insular Asia, in Indonesia (Sumatra) and East Malaysia (Sabah) and from Continental Asia, in west Malaysia (Pahang) and Thailand (Songkla and Narathiwat) (Bae and McCafferty 1991; Parnrong et al. 2002; Kwanboon et al. 2021). The most diverse family in the Oriental region, Baetidae, also has a widespread species, *Labiobaetismoriharai* Müller-Liebenau, 1984. This species was recorded in Insular Asia in Brunei (Temborong) and Malaysia (Sabah) and in Continental Asia in Malaysia (Selangor) and Vietnam (Dong Nai) (Kaltenbach and Gattolliat 2020). The distribution of *P.campylociella* is limited to eastern Malaysia and the distribution of the type species *P.vitalisi* is limited to the type locality (Tonkin, Vietnam); the nymph remains a mystery.

Our study allows us to conclude that the genus *Polyplocia* in Thailand is represented by two species, *P.orientalis* and *P.nebulosa*, based on a combination of different morphological characters, egg characters and molecular evidence. We have reported the first nominal species records of this family in Thailand and expanded the distribution of these two species. The distribution of *P.orientalis* in Thailand is limited to the northern parts and mainland Asia, while the distribution of *P.nebulosa* extends from Insular Asia to Continental Asia, in western Thailand in our study. We expect to find a broader distribution of this genus in Thailand, especially in the southern and the eastern parts, as we gain a more in-depth understanding of the distribution pattern of this genus in the Oriental region.

## Supplementary Material

XML Treatment for Polyplocia

XML Treatment for Polyplocia vitalisi

XML Treatment for Polyplocia campylociella

XML Treatment for Polyplocia nebulosa

XML Treatment for Polyplocia orientalis

## ﻿Additional information

### Author ORCIDs

Sedtawut Kwanboon https://orcid.org/0000-0002-3858-9522

Boonsatien Boonsoong https://orcid.org/0000-0002-8166-0021

Chanaporn Suttinun https://orcid.org/0000-0002-0380-1554

### Data availability

All of the data that support the findings of this study are available in the main text.
